# Evaluation of platelet surface-associated immunoglobulin positivity and its association with hematologic findings and vector-borne pathogens in thrombocytopenic dogs

**DOI:** 10.1093/jvimsj/aalag033

**Published:** 2026-03-06

**Authors:** Warattha Boontuboon, Walasinee Sakcamduang, Rungrote Osathanon, Teerawut Nedumpun, Paitoon Srimontri

**Affiliations:** Department of Clinical Science and Public Health, Faculty of Veterinary Science, Mahidol University, 999 Phutthamonthon Sai 4 Road, Salaya, Phutthamonthon, Nakhon Pathom 73170, Thailand; Department of Clinical Science and Public Health, Faculty of Veterinary Science, Mahidol University, 999 Phutthamonthon Sai 4 Road, Salaya, Phutthamonthon, Nakhon Pathom 73170, Thailand; Department of Clinical Science and Public Health, Faculty of Veterinary Science, Mahidol University, 999 Phutthamonthon Sai 4 Road, Salaya, Phutthamonthon, Nakhon Pathom 73170, Thailand; Department of Veterinary Microbiology, Faculty of Veterinary Science, Chulalongkorn University, 39 Henri-Dunant Road, Wangmai, Pathumwan, Bangkok 10330, Thailand; Department of Clinical Science and Public Health, Faculty of Veterinary Science, Mahidol University, 999 Phutthamonthon Sai 4 Road, Salaya, Phutthamonthon, Nakhon Pathom 73170, Thailand

**Keywords:** immune thrombocytopenia, ITP, PSAIG, *Ehrlichia canis*

## Abstract

**Background:**

Platelet surface–associated immunoglobulin (PSAIG) occurs in thrombocytopenic dogs with vector-borne diseases and immune thrombocytopenia (ITP) and may be associated with thrombocytopenia severity and inflammatory markers, including neutrophil-to-lymphocyte and platelet-to-lymphocyte ratios (NLR, PLR).

**Hypothesis/Objectives:**

Assess associations between PSAIG positivity, hematologic parameters, thrombocytopenia severity, and vector-borne status in thrombocytopenic dogs.

**Animals:**

Sixty-nine client-owned thrombocytopenic dogs (<200 × 10^3^/μL) were enrolled between June 2022 and June 2023.

**Methods:**

Dogs were prospectively enrolled. Platelet surface–associated immunoglobulin was measured using flow cytometry. Vector-borne pathogens were assessed by serology (*Ehrlichia* spp., *Anaplasma* spp., *Borrelia burgdorferi, Dirofilaria immitis*) and PCR for *Ehrlichia canis*. Hematologic parameters were compared between PSAIG groups (Mann–Whitney *U*), and associations tested by univariable logistic regression.

**Results:**

Dogs positive for PSAIG (*n* = 16) had lower median automated platelet counts (16.5 × 10^3^/μL; interquartile range [IQR]: 8.25-40.75) than PSAIG-negative dogs (*n* = 53; 64 × 10^3^/μL; IQR: 25.0-92.5; *P* = .001), with similarly lower manual platelet counts (48 × 10^3^/μL; IQR: 20-86 vs 96 × 10^3^/μL; IQR: 55–138; *P* = .01) and automated PLR (7.14; IQR: 3.30-15.28 vs 21.82; IQR: 9.42–38.99; *P* = .01). In logistic regression, PSAIG positivity was associated with lower platelet counts and automated PLR, *E. canis* PCR positivity, and *Anaplasma* seropositivity, with the strongest association for concurrent *E. canis* PCR and *Anaplasma* seropositivity (odds ratio [OR]; 15.3; 95% confidence interval [CI]: 2.69-86.99; *P* = .002).

**Conclusions and clinical importance:**

Lower platelet counts and automated PLR were associated with PSAIG positivity in thrombocytopenic dogs. Associations between PSAIG, *E. canis* infection, and co-exposure to *Anaplasma* spp. support immune-mediated platelet destruction in infected dogs.

## Introduction

Immune thrombocytopenia (ITP) is the most commonly observed acquired primary hemostatic disorder in dogs.^1^ The pathogenesis of ITP includes several immune-mediated mechanisms involving humoral and cell-mediated immunity,^2^ and inappropriately low thrombopoietin (TPO) concentrations.^3^ The disease is considered primary (non-associative) when no underlying cause is identified, and secondary (associative) when ITP results from stimulation by other factors such as infections, systemic inflammation, neoplasia,^4^ and certain medications such as sulfonamides and cephalosporins.^5,6^

The detection of platelet surface–associated immunoglobulin (PSAIG) suggests that an immune-mediated mechanism may be involved, but it is neither definitive for diagnosing ITP^7^ nor capable of distinguishing between primary and secondary forms.^8,9^ It can also be detected in dogs with non-immune causes of thrombocytopenia, such as infectious, neoplastic, or inflammatory diseases.^7^ Therefore, its presence alone does not confirm that immune-mediated platelet destruction is responsible for thrombocytopenia in a given patient. Detection of PSAIG, however, can provide supportive evidence for ITP after exclusion of other causes of thrombocytopenia. Severe thrombocytopenia is common in dogs with ITP, but it is not unique to the disorder.^4,7,8,10^ Positivity for PSAIG and platelet counts <20 × 10^3^/μL perform similarly in differentiating primary ITP from other causes of thrombocytopenia.^7^ To our knowledge, studies evaluating whether PSAIG positivity correlates with the magnitude of thrombocytopenia have not been performed; establishing such an association could help clarify its clinical and pathophysiologic relevance. Platelet-to-lymphocyte ratio (PLR) and neutrophil-to-lymphocyte ratio (NLR) have emerged as markers in a range of conditions in both veterinary and human medicine. In human medicine, higher PLR has been associated with a lower risk of glucocorticoid resistance^11^ and decreased recurrence in ITP,^12^ highlighting its potential prognostic value. Increased PLR has also been observed in several diseases, including pancreatitis in dogs and cats,^13^ and in dogs with non-septic disease or parvoviral infection, where nonsurvivors exhibited significantly higher PLR than survivors.^14,15^ Despite these findings, the relationship between PLR and PSAIG positivity has not been explored in dogs. Establishing such an association could be clinically relevant because PLR, readily calculated from the CBC, might serve as an inexpensive, indirect marker to suggest that PSAIG may be present.

Infections reported to be associated with the occurrence of platelet-bound antibodies in dogs include *Ehrlichia canis*,^4,8,16–18^  *Leishmania infantum,*^4,19^  *Anaplasma phagocytophilum*,^4,8,20^ and *Babesia* spp.^4,16^ Although prior studies have identified associations between PSAIG positivity and vector-borne pathogens, to our knowledge, none have quantified the strength of these associations, particularly using odds ratios (ORs). Therefore, we aimed to provide quantitative insight into this relationship. Our objectives were to assess the presence and strength of associations between PSAIG and selected hematologic parameters, the magnitude of thrombocytopenia, and vector-borne pathogen status in thrombocytopenic dogs.

## Materials and methods

### Animals

Client-owned thrombocytopenic dogs receiving veterinary care at the Prasu Arthorn Veterinary Teaching Hospital, Faculty of Veterinary Science, Mahidol University, Thailand, between June 2022 and June 2023 were studied. Thrombocytopenia was defined as a platelet count <200 × 10^3^/μL without evidence of platelet clumping.^21^ Hematologic reports were reviewed by one of the authors (W.B.), who screened dogs for eligibility based on inclusion and exclusion criteria. Dogs were included if they were at least 3 months of age, had not received doxycycline, minocycline, glucocorticoids, or any immunosuppressive drugs before the study, and there were no restrictions on sex or body weight. Dogs were excluded if they were Greyhounds or Cavalier King Charles Spaniels (because of potential confounding factors such as lower platelet count and hereditary macrothrombocytopenia, respectively^22^). Dogs were enrolled prospectively, and data were collected at the time of presentation. After completion of enrollment, data were reviewed, and dogs with incomplete medical records or missing CBC results were excluded before statistical analysis. For analysis, diagnoses were categorized into the following groups: Neoplastic disease was diagnosed based on histopathologic or cytologic evaluation. Inflammatory or infectious disease was identified based on clinical signs, diagnostic imaging, and laboratory evaluation, including CBC, cytology, and additional disease-specific tests as clinically indicated (eg, pancreatic lipase immunoreactivity for pancreatitis, bacterial culture for abscesses). These cases were grouped together to reflect systemic or localized inflammation or infection not related to vector-borne pathogens. Vector-borne diseases were analyzed separately because of their specific relevance to PSAIG. Diagnostic testing for vector-borne pathogens is described in the vector-borne infection section of the Materials and methods. All dogs underwent a standardized diagnostic evaluation that included CBC, vector-borne disease screening, and imaging (thoracic and abdominal radiographs or ultrasonography). Cytology or histopathology was performed as clinically indicated to identify or exclude potential underlying causes of thrombocytopenia. Dogs were classified as having probable primary immune thrombocytopenia (pITP) with and without immunologic evidence according to an American College of Veterinary Internal Medicine (ACVIM) Consensus Statement,^7^ and only after diagnostic evaluation excluded other likely causes of thrombocytopenia and potential underlying diseases associated with secondary (associative) ITP. Thrombocytopenia was confirmed by manual platelet count on a blood smear. Causes of platelet consumption or loss, such as hemorrhage, disseminated intravascular coagulation (DIC), or sepsis were assessed based on clinical history, physical examination, and laboratory results. No additional unexplained cytopenias (leukopenia or anemia) were present. Partial screening for potential trigger factors, including vector-borne diseases (*Ehrlichia* spp., *Anaplasma* spp., and *Babesia* spp.) and neoplasia, was performed using infectious disease testing and diagnostic imaging. A diagnosis of pITP additionally required sustained platelet count recovery after immunosuppressive treatment, defined as an increase in platelet count to ≥100 × 10^3^/μL, and cases were further classified as pITP with immunologic evidence when PSAIG positivity was documented. The study protocol was approved by the Mahidol University-Institute Animal Care and Use Committee of the Faculty of Veterinary Science, under protocol number MUVS-2022-0206.

### Hematology

Procedures were conducted on the respective days of sample collection. Blood samples of 1-2 mL were collected in EDTA tubes for hematologic assessment, performed using a Mindray BC 5300 Vet analyzer (Mindray, Guangdong, China), along with manual platelet counting. Platelet margination was assessed by reviewing the smear edges, and platelet count was determined by averaging the count from 10 oil immersion fields (100×) on the blood film monolayer. The manual platelet count (per μL) was calculated as follows: Manual platelet count (/μL) = average platelets/field × 15 000.^22^ The NLR and PLR were calculated by dividing the neutrophil and platelet counts, respectively, by the lymphocyte count.

### Vector-borne infection

Each dog underwent testing for *Ehrlichia* spp., *Anaplasma* spp., and *Borrelia burgdorferi* antibodies, as well as *Dirofilaria immitis* antigens, using commercially available kits (SNAP 4Dx Plus IDEXX and VETSCAN Flex4 Zoetis platforms). Additionally, each dog was screened for the presence of DNA from *E. canis, Babesia* spp., and *Hepatozoon canis* using PCR. The multiplex PCR primers were specific to *E. canis* VirB9, *Babesia* spp. 16S rRNA and *H. canis* 16S rRNA genes according to protocols previously described in the literature.^23,24^ Testing was conducted at The Monitoring and Surveillance Center for Zoonotic Diseases in Wildlife and Exotic Animals, Faculty of Veterinary Science, Mahidol University.

### Direct flow cytometric assay for platelet surface-associated immunoglobulin

The remaining EDTA blood samples were submitted to the Chulalongkorn University-Veterinary Diagnostic Laboratory (CU-VDL) at the Faculty of Veterinary Science, CU, for analysis using a direct flow cytometric assay specifically developed to detect PSAIG.^25^ Briefly, whole blood samples were stored at 4 °C before processing, with a storage duration of <24 hours. Whole blood samples were centrifuged at 150 × *g* for 15 minutes. The platelet-rich fraction (PRF) was isolated for further centrifugation at 850 × *g* for 10 minutes. The pellet underneath the plasma was collected for immunofluorescent staining. To identify the platelet population, the pellet was stained using anti-sheep CD41/61 (platelet marker) monoclonal antibody (Ab) (mouse IgG1, Bio-Rad, United States). Fluorescein isothiocyanate-conjugated anti-canine IgG monoclonal Ab (goat IgG, Bio-Rad) also was included in the staining cocktail. The cells then were incubated in the dark at 4 °C for 20 minutes. The stained cells were analyzed using a Beckman Coulter FC500 and CytoFlex LX. The positive cells were referred to IgG-coated platelets or PSAIG. The cut-off value for IgG-coated platelets was established in-house at VDL, Faculty of Veterinary Science, Chulalongkorn University (CU-VDL). A 10% threshold of IgG-coated platelets was applied to confirm PSAIG positivity. This finding is consistent with previous studies,^19,26,27^ which identified >10% IgG-coated platelets as a valid criterion for PSAIG positivity. In our study, the EDTA blood samples obtained from ITP-suspected dogs were processed and subjected to immunofluorescent labeling for CD41/61 and IgG, as described earlier. The presence of PSAIG was considered if dogs showed significantly higher numbers of IgG-coated platelets than the cut-off value.

### Statistical analysis

Statistical analysis was performed using Statistical Package for the Social Sciences (SPSS) version 23 for Windows (SPSS, Chicago, IL). The threshold for statistical significance was set at *P* < .05. Descriptive statistics were used to summarize the study population, with median and range for continuous variables and proportions for categorical data. The Mann–Whitney *U* test was applied to compare hematologic parameters between PSAIG-positive and PSAIG-negative groups. Univariable logistic regression analysis was conducted to identify factors associated with PSAIG positivity, estimating OR with 95% CIs. The model fit was assessed using the Hosmer-Lemeshow test.

## Results

### Study population

Eighty-seven dogs with thrombocytopenia were identified and included in the study. However, 18 were excluded because of incomplete hematologic data (13) and absence of a diagnosis (5). As a result, 69 dogs met the inclusion criteria. Among the 69 dogs with thrombocytopenia, 2 dogs (2.9%) were diagnosed as probable pITP: one classified as probable pITP and the other as probable pITP with immunologic evidence. Because laboratory evaluation for DIC and other secondary hemostasis disorders was not conducted in these dogs, their classification as pITP was based on exclusion of other potential causes of thrombocytopenia and a sustained platelet count recovery after immunosuppressive treatment. None of these dogs exhibited clinical evidence of vasculitis or sepsis. Among the remaining 67 dogs, 20 (28.89%) had vector-borne infection or exposure without comorbid conditions; 23 (33.33%) had neoplasia including carcinoma (*n* = 5), tumor of epithelial origin (*n* = 4), lymphoma (*n* = 3), melanoma (*n* = 2), and one each of Sertoli cell tumor, transmissible venereal tumor (TVT), myelolipoma, and myoepithelioma; 14 (20.29%) had inflammatory or infectious diseases including acute pancreatitis (*n* = 2), pyometra (*n* = 2), cholangiohepatitis (*n* = 2) and one each of suppurative splenitis, superficial bacterial folliculitis, allergic skin disease, myositis, renal abscess, chronic bronchitis, pneumonia, and chronic inflammatory enteropathy; and, 10 (14.49%) had miscellaneous disorders, including cryptorchidism (*n* = 2), diabetes mellitus (*n* = 2), and one each of ovarian cyst, intestinal foreign body, chordae tendineae rupture, myxomatous mitral valve disease (MMVD) stage B2, chronic kidney disease (CKD), and cystic calculi. Cases were further classified based on concurrent blood parasite infections into neoplasia, inflammatory or infectious disease, and miscellaneous disorder groups (Table 1).

**Table 1:** Results of platelet surface-associated immunoglobulin (PSAIG) using flow cytometric assay in 69 thrombocytopenia dogs with various diseases and vector-borne infections.

**Diseases**	**Blood parasites**	***N***	**PSAIG**	
			**Positive**	**Negative**
**Neoplasia**	*Ehrlichia canis* PCR positive	2	0	2
	*Ehrlichia* spp. seropositive	3	0	3
	*Anaplasma* spp. seropositive	1	0	1
	*Ehrlichia* spp. and *Anaplasma* spp. seropositive	2	0	2
	*Ehrlichia* spp., *Anaplasma* spp. seropositive and *E.canis* PCR positive	2	1	1
	*Babesia* spp. PCR positive	1	0	1
	No vector-borne pathogen infection/exposure	12	3	9
**Inflammatory/infectious disease**	*Ehrlichia* spp. and *Anaplasma* spp. seropositive	2	0	2
	*Ehrlichia* spp., *Anaplasma* spp. seropositive and *E.canis* PCR positive	1	1	0
	No vector-borne pathogen infection/exposure	11	1	10
**Miscellaneous diseases**	*Ehrlichia canis* PCR positive	1	0	1
	*Ehrlichia* spp. seropositive	1	0	1
	*Ehrlichia canis* PCR positive and *Ehrlichia* spp. seropositive	1	0	1
	*Anaplasma* spp. seropositive	2	1	1
	*Babesia* spp. PCR positive	1	0	1
	No vector-borne pathogen infection/exposure	4	0	4
**Vector-borne infection/exposure**	*Ehrlichia canis* PCR positive	2	1	1
	*Ehrlichia* spp. seropositive	1	0	1
	*Ehrlichia canis* PCR positive and *Ehrlichia* spp. seropositive	2	1	1
	*Ehrlichia* spp. and *Anaplasma* spp. seropositive	3	0	3
	*Ehrlichia* spp., *Anaplasma* spp. seropositive and *E.canis* PCR positive	4	3	1
	*Ehrlichia* spp., *Anaplasma* spp. seropositive, *E.canis* and *Babesia* spp. PCR positive	1	1	0
	*Ehrlichia* spp. seropositive, *E.canis* and *Babesia* spp. PCR positive	1	0	1
	*Ehrlichia canis* and *Babesia* spp. PCR positive	2	1	1
	*Ehrlichia canis* and *H. canis* PCR positive	1	0	1
	*Ehrlichia canis, H. canis*, and *Babesia* spp. PCR positive	1	0	1
	*Anaplasma* spp. seropositive and *Babesia* spp. PCR positive	2	1	1
**Probable primary ITP**	-	2	1	1

Flow cytometric analysis identified 16 dogs as PSAIG-positive and 53 dogs as PSAIG-negative. Among the PSAIG-positive group, seven distinct breeds were represented, including 9 crossbreeds and 7 purebred dogs (comprising 2 Poodles and one each of Pomeranian, Alaskan Malamute, Chihuahua, Bangkaew, and American Bully). In the PSAIG-negative group, 16 breeds were observed, consisting of 19 crossbreeds and 34 purebred dogs (including 6 Poodles, 6 Bangkaew, 5 Pomeranian, 3 Shih Tzus, 2 Siberian Huskies, 2 Chihuahuas, 2 Labrador Retrievers, and one each of Golden Retriever, American Bully, Maltese, Boxer, Miniature Pinscher, Pekingese, American Cocker Spaniel, and Corgi). The PSAIG-positive dogs had a median age of 6.5 years (interquartile range [IQR: 2.50-10.00]), whereas the PSAIG-negative dogs had a median age of 9 years (IQR: 5.00-12.00). There were nine males and seven females in the PSAIG-positive group and 26 males and 27 females in the PSAIG-negative group. The median weight of dogs diagnosed as PSAIG-positive was 17.95 kg (IQR: 9.15-24.65), and the PSAIG-negative dogs had a median weight of 15.7 kg (IQR: 6.10-23.48).

### Hematologic analysis

**Figure 1:**
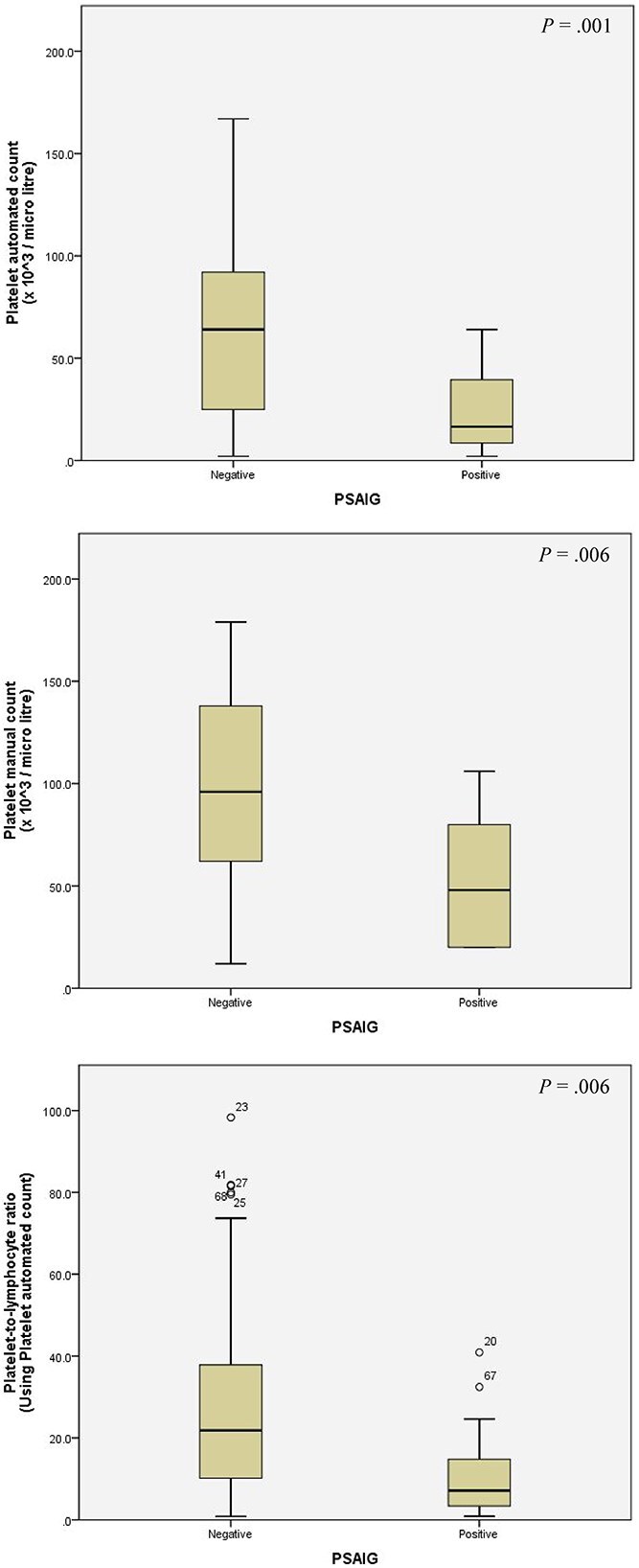
Box plots of variables associated with platelet surface-associated immunoglobulin (PSAIG) using flow cytometric assay according to the univariable logistic regression analysis.

The hematologic parameters were compared between PSAIG-positive and negative groups using a Mann–Whitney *U* test, the details of which are shown in Table 2. The PSAIG-positive dogs (*n* = 16) had significantly lower median automated platelet counts (median: 16.50 × 10^3^/μL; IQR: 8.25-40.75) compared with PSAIG-negative dogs (*n* = 53; median: 64 × 10^3^/μL; IQR: 25.00-92.50; *P* = .001). Manual platelet counts were also lower in PSAIG-positive dogs (median: 48 × 10^3^/μL; IQR: 20-86) versus PSAIG-negative dogs (median: 96 × 10^3^/μL; IQR: 55-138; *P* = .006). The PLR calculated using the automated platelet count was also significantly lower in PSAIG-positive dogs (median: 7.14; IQR: 3.30-15.28) compared with PSAIG-negative dogs (median: 21.82; IQR: 9.42-38.99; *P* = .01). The box plot illustrates the median, IQR, and any outliers for each group (Figure 1). No significant differences were observed in other hematologic parameters between the PSAIG-positive and PSAIG-negative groups.

**Table 2:** Comparison of dog characteristics and hematological values based on platelet surface-associated immunoglobulin (PSAIG) detection using flow cytometric assay.

**Factor**	**Variable**	**PSAIG**		***P* value**
		**Positive (*n* = 16)**	**Negative (*n* = 53)**	
**Characteristic**	Age (years)	6.5 [2.50-10.00]	9 [5.00-12.00]	.10
	Sex (M/F) (%)	(9/7) (56.25/43.75)	26/27 (49.00/51.00)	.6
	Breed (pure/mixed) (%)	(7/9) (43.75/56.25)	(34/19) (64.20/35.80)	.15
	Body weight (kg)	17.95 [9.15-24.65]	15.7 [6.10-23.48]	.4
**Hematology**	WBC (10^3^/μL)	9.79 [7.88-18.36]	12.33 [8.88-18.76]	.3
	Monocyte (10^3^/μL)	0.36 [0.24-0.67]	0.43 [0.32-0.85]	.2
	Neutrophil (10^3^/μL)	7.48 [5.9-14.14]	8.58 [5.48-13.73]	.7
	Lymphocyte (10^3^/μL)	2.28 [1.48-2.65]	2.42 [1.82-3.24]	.3
	Eosinophil (10^3^/μL)	0 [0-0.10]	0 [0-0.36]	.19
	Basophil (10^3^/μL)	0 [0-0]	0 [0-0]	1
	Band neutrophil (10^3^/μL)	0 [0-0.30]	0 [0-0.0005]	.9
	NLR	4.12 [3.14-4.92]	3.70 [3.02-4.75]	.5
	RBC (10^6^/μL)	5.65 [4.0-6.0]	5.76 [4.15-6.56]	.6
	Hb (g/dL)	13.20 [9.05-14.28]	12.90 [9.65-14.75]	.8
	HCT (%)	39.20 [27.38-43.30]	38.80 [29.20-44.30]	.8
	MCV (fL)	69.20 [66.55-73.73]	69.30 [65.65-72.70]	.6
	MCH (pg)	23.45 [22.28-24.58]	23.40 [22.25-24.15]	.5
	MCHC (pg)	33.50 [32.70-34.78]	33.40 [32.45-34.25]	.8
	RDW (%)	13.55 [12.28-15.78]	13.20 [12.30-14.45]	.7
	Platelet automated count (10^3^/μL)	16.50 [8.25-40.75]	64 [25.00-92.50]	.001
	Platelet manual count (10^3^/μL)	48 [20-86]	96 [55-138]	.01
	PLR (Platelet automated count)	7.14 [3.30-15.28]	21.82 [9.42-38.99]	.006
	PLR (Platelet manual count)	20.49 [13.15-35.80]	31.38 [19.74-52.92]	.08

Abbreviations: [IQR] WBC = white cell count; NLR = neutrophil-to-lymphocyte ratio; RBC = red blood cell count; Hb = hemoglobin; HCT = hematocrit; MCV = mean corpuscular volume; MCH = mean corpuscular hemoglobin; MCHC = mean corpuscular hemoglobin concentration; RDW = red cell distribution width; PLR = platelet-to-lymphocyte ratio; IQR = interquartile range.

### Distribution of PSAIG positivity by disease category and vector-borne infection status

The PSAIG positivity was evaluated across various disease categories and vector-borne infections in dogs (Table 1). Among dogs diagnosed with neoplasia (*n* = 23), 4 dogs were PSAIG-positive, and 19 were PSAIG-negative. Among the PSAIG-positive dogs, one dog had concurrent *E. canis* infection confirmed by PCR and seropositivity for *Anaplasma* spp. and *Ehrlichia* spp.; this dog was diagnosed with a tumor of epithelial origin on the trunk based on cytologic evaluation. The remaining 3 PSAIG-positive dogs with neoplasia had no detectable vector-borne infection or exposure. These dogs were diagnosed with TVT at the bulbus glandis (by cytology), fibrosarcoma of the left eye (by histopathology), and Sertoli cell tumor (by histopathology). In the inflammation group (*n* = 14), only one dog with no vector-borne infection tested PSAIG-positive; this dog was diagnosed with cholangiohepatitis. One dog with concurrent *E. canis* infection and seropositivity for *Anaplasma* spp. and *Ehrlichia* spp. was also PSAIG-positive; this dog was diagnosed with superficial bacterial folliculitis. Among those with miscellaneous diseases (*n* = 10), 1 dog was PSAIG-positive. This dog was also seroactive to *Anaplasma* spp. In the vector-borne infection group (*n* = 20), 8 dogs were PSAIG-positive. Three of these dogs were PCR positive for *E. canis* and seroreactive to both *Anaplasma* and *Ehrlichia* spp. Positivity for PSAIG was documented in one dog with *E. canis* PCR positivity, one dog with both *E. canis* PCR positivity and *Ehrlichia* spp. seropositivity, one dog seropositive for *Ehrlichia* spp. and *Anaplasma* spp. with concurrent *E. canis* and *Babesia* spp. PCR positivity, one dog with *E. canis* and Babesia spp. PCR positivity, and one dog seropositive for *Anaplasma* spp. with *Babesia* spp. PCR positivity. No dogs had detectable *B. burgdorferi* antibodies or *D. immitis* antigens.

### Factors associated with PSAIG positivity

Univariable logistic regression analysis identified three hematological variables significantly associated with PSAIG positivity: lower platelet counts by both automated and manual methods, and a lower PLR based on automated counts (Table 3). In the context of vector-borne pathogens, univariable logistic regression identified three significant predictors of PSAIG positivity among thrombocytopenic dogs. Dogs with *E. canis* infection confirmed by PCR, whether alone or in combination with other vector-borne pathogens, had an OR of 4.39 (95% CI: 1.35-14.28; *P* = .01) for being PSAIG-positive. Similarly, dogs with *Anaplasma* spp. seropositivity, either alone or with other pathogens, had an OR of 3.42 (95% CI: 1.06-11.03; *P* = .04). The strongest association was observed in dogs concurrently with at least *E. canis* PCR positivity and *Anaplasma* spp. seropositivity, with an OR of 15.3 (95% CI: 2.69-86.99; *P* = .002). Other vector-borne infection or exposure categories, including *Babesia* spp. and *H. canis*, did not show significant associations or yielded unstable estimates because of sparse data or the absence of PSAIG-positive cases.

**Table 3:** Univariable logistic regression analysis of factors associated with platelet surface-associated immunoglobulin (PSAIG) using flow cytometric assay.

**3.1 Hematological variables**			
**Variables**	**Odds ratio**	**95% CI of the odds ratio**	***P* value**
**Age (year)**	0.8	(0.76-1.02)	.09
**Body weight (kg)**	1.02	(0.98-1.08)	.3
**WBC**	1.00	(1.00-1.00)	.3
**Monocyte**	1.00	(1.00-1.07)	.7
**Neutrophil**	1.00	(1.00-1.00)	.5
**Lymphocyte**	1.00	(1.00-1.00)	.3
**Eosinophil**	0.99	(0.99-1.00)	.2
**Band neutrophil**	1.00	(1.00-1.00)	.6
**NLR**	1.06	(0.80-1.41)	.7
**RBC**	0.90	(0.65-1.26)	.6
**Hb**	0.96	(0.84-1.11)	.7
**HCT**	0.98	(0.94-1.04)	.6
**MCV**	1.01	(0.90-1.14)	.8
**MCH**	0.97	(0.85-1.12)	.7
**MCHC**	0.97	(0.80-1.18)	.8
**RDW**	1.09	(0.79-1.52)	.6
**Platelets automated count (× 10^3^/μL)**	0.96	(0.94-0.99)	.004
**Platelets manual count (× 10^3^/μL)**	0.98	(0.97-0.99)	.01
**PLR (Platelets automated count)**	0.94	(0.89-0.99)	.02
**PLR (Platelets manual count)**	0.97	(0.95-1.00)	.08
**3.2 Presence of vector-borne pathogens**			
**Variables (At least)**	**Odds ratio**	**95% CI of the odds ratio**	***P* value**
***E.canis* PCR positive**	4.39	(1.35-14.28)	.01
***E.canis* PCR positive or *Ehrlichia* spp. seropositive**	1.55	(0.504-4.791)	.4
***Anaplasma* spp. seropositive**	3.42	(1.06-11.03)	.04
***E.canis* PCR positive or *Ehrlichia* spp. seropositive and *Anaplasma* spp. seropositive**	0.34	(0.10-1.18)	.09
***E.canis* PCR positive and *Anaplasma* spp. seropositive**	15.3	(2.69-86.99)	.002
***Babesia* spp. PCR positive**	2.61	(0.63-10.75)	.18
***Hepatozoon canis* PCR positive^a^**	NA	NA	.9
***E.canis* and *Babesia* spp. PCR positive**	2.38	(0.36-15.68)	.36
***E.canis* PCR positive or *Ehrlichia* spp. seropositive and *Babesia* spp. PCR positive**	2.38	(0.36-15.68)	.36
***Anaplasma* spp. seropositive and *Babesia* spp. PCR positive**	7.43	(0.63-87.99)	.11
***E.canis* and *H. canis* PCR positive^a^**	NA	NA	.9
***E.canis* PCR positive or *Ehrlichia* spp. seropositive and *H. canis* PCR positive^a^**	NA	NA	.9

Abbreviations: WBC = white cell count; NLR = neutrophil-to-lymphocyte ratio; RBC = red blood cell count; Hb = hemoglobin; HCT = hematocrit; MCV = mean corpuscular volume; MCH = mean corpuscular hemoglobin; MCHC = mean corpuscular hemoglobin concentration; RDW = red cell distribution width; PLR = platelet-to-lymphocyte ratio.

^a^Result could not be estimated due to zero events.

## Discussion

In our study, *E. canis* infection, particularly in conjunction with seroreactivity to *Anaplasma* spp, was strongly associated with PSAIG. Lower platelet counts and PLR (using automatic platelet counts) also were associated with PSAIG.

Among 69 dogs with thrombocytopenia, the most common diagnoses considered to be the primary cause of thrombocytopenia were neoplasia (*n* = 23; 33.3%), vector-borne infection or exposure (*n* = 20; 29.0%), other inflammatory or infectious diseases (*n* = 14; 20.3%), miscellaneous diseases (*n* = 10; 14.5%), and pITP (*n* = 2; 2.9%), of which one was classified as probable pITP and the other as probable pITP with immunologic evidence. Evidence of vector-borne disease was detected in 40 dogs (58.0%), including 20 dogs in which vector-borne infection was identified as the principal cause of thrombocytopenia, as well as 11 dogs with concurrent neoplasia, 3 with inflammatory or infectious disease, and 6 with miscellaneous diseases. This distribution aligns with findings from a previous study,^28^ which investigated 871 dogs with thrombocytopenia and reported inflammatory or infectious diseases, including dogs positive for blood parasite Ab or antigen tests, as the most common cause (34.9%), followed by neoplasia (23%), miscellaneous disorders (25.5%), DIC (6%), and pITP (5.6%). In contrast, a more recent study of 762 dogs with thrombocytopenia found that neoplasia was the most prevalent associated condition (27.3%), followed by miscellaneous causes (26.9%), pITP (18.8%), inflammatory or immune-mediated disorders (14.4%), and infectious diseases (12.6%).^29^ The variation in these findings may be attributed to differences in disease prevalence across geographic regions, particularly given that our study was conducted in an area endemic for tick-borne diseases.^30^

The vector-borne infectious agents tests conducted in our study included the serologic test for *A. phagocytophilum/Anaplasma platys, E. canis/Ehrlichia ewingii, Ehrlichia chaffeensis*, and *B. burgdorferi*. The presence of DNA from *E. canis, Babesia* spp., and *H. canis* was tested using a multiplex PCR. According to a recent ACVIM consensus statement on the diagnosis of ITP, there is a high level of evidence that *E. canis* can cause ITP in dogs.^7^ Platelet surface-associated immunoglobulin forms during infection, suggesting that immune-mediated platelet destruction may contribute to thrombocytopenia.^18,19,31,32^ In the same consensus statement, intermediate levels of evidence were found for *Anaplasma* spp. as a cause of ITP in dogs.^7^ In our study population, it was not feasible to statistically assess the effect of individual vector-borne pathogens because of the presence of frequent coinfections or coexposures. Notably, dogs with PCR-confirmed of *E.canis* infection also seroreactive for *Anaplasma* spp. had the highest OR of PSAIG positivity (OR, 15.3; 95% CI: 2.69-86.99; *P* = .002) compared with dogs without this co-infection. This finding suggests a possible relationship between co-infection and increased PSAIG production. However, a limitation of our study is that *Anaplasma* spp. was only assessed using serology detection rather than PCR. The presence of *Anaplasma* spp. antibodies indicateprevious exposure and not necessarily ongoing infection, which complicates the interpretation of our findings.^33^ Moreover, serologic testing may be negative during acute infection before Ab development, meaning a negative result does not definitively exclude current infection. Additionally, previous studies have highlighted the potential for serological cross-reactivity between *E. canis* and *A. phagocytophilum*,^34^ raising concerns about the specificity of Ab-based detection methods. This cross-reactivity could lead to false-positive *Anaplasma* spp. serology results, further complicating the interpretation of our findings. Further investigation is needed to determine whether immune-mediated platelet destruction occurs in association with *A. platys* infection, which is transmitted by *Rhipicephalus sanguineus*,^34^ the same vector as *E. canis*. Both pathogens have been reported in dogs in Thailand.^30^ Currently, there is a low level of evidence that *Babesia* spp. cause ITP, but properly designed studies are needed to further explore whether immune-mediated destruction contributes to thrombocytopenia in infected dogs.^7^ Notably, none of the dogs infected with *Babesia* spp. in the absence of coinfection tested positive for PSAIG. However, PSAIG was observed in dogs infected with *Babesia* spp. coinfected with *E. canis* or seroreactive to *Anaplasma* spp. However, the association appears inconsistent because PSAIG was not demonstrated in all coinfected dogs.

Our study did not include testing for *Leishmania* spp., despite high-level evidence indicating that *L. infantum* infection is a known cause of ITP in dogs.^19,35,36^ This decision was based on the fact that Thailand is considered a non-endemic region for leishmaniasis, although sporadic cases have been reported in humans, particularly in the southern part of the country.^37–39^ Additionally, previous research has documented a low seroprevalence of *Leishmania* spp. infection in both dogs and cats in Thailand.^40^ Our study was conducted in the central region of Thailand, and all dogs enrolled were from that region.

Currently, the overall evidence supporting neoplasia as a cause of ITP in dogs is limited to negligible, with insufficient data to establish a causal link between specific tumor types and ITP.^7^ This evidence grading is a consequence of the paucity of studies and the lack of robustly designed investigations, rather than definitive evidence ruling out a causal relationship. In our study, PSAIG positivity was observed in dogs with neoplasia and no detectable vector-borne infection or exposure, including cases of TVT, Sertoli cell tumor, and fibrosarcoma of the eye. Although ITP has not been directly documented in dogs with TVT, previous studies have reported thrombocytopenia as part of the hematologic profile,^41,42^ suggesting a possible paraneoplastic or inflammatory mechanism. Further research is needed to determine whether immune processes contribute to thrombocytopenia in TVT. Sertoli cell tumors are known to cause thrombocytopenia through estrogen-induced bone marrow suppression,^43,44^ but there is currently no evidence linking them to immune-mediated mechanisms. Notably, one dog with a Sertoli cell tumor in our study was PSAIG-positive, raising the possibility of an immune-mediated component in selected cases. Similarly, fibrosarcoma was identified as PSAIG-positive in one dog. A single case of ITP has been reported in a dog with a poorly differentiated fibrosarcoma.^45^ Our finding aligns with this observation and suggests that ITP may occur in association with certain solid tumors.

A platelet count <20 × 10^3^/μL supports a diagnosis of ITP.^46^ Our study identified a significant inverse relationship between the likelihood of being PSAIG-positive and decreased platelet counts, as documented by automated and manual counts. Specifically, for every decrease of 1,000 in automated platelet count, the likelihood of being PSAIG-positive increased by 3.4%. Similarly, for every decrease of 1000 in manual platelet count, the possibility of being PSAIG-positive increased by 2%. In addition, a significant decrease in PLR using automated platelet counts was observed among dogs positive for PSAIG compared with those without. A one-unit decrease in PLR was associated with a 5% increase of PSAIG positivity. Increased PLR has been noted in various diseases, such as pancreatitis, in dogs and cats.^13^ Moreover, in dogs with non-septic diseases, nonsurvivors had notably higher median PLR than survivors.^14^ Similarly, among dogs with parvovirus infection, nonsurvivors had significantly higher median PLR than survivors.^15^Although specific investigations in dogs with ITP are limited, studies in humans suggest that PLR may have prognostic value in ITP. One study determined that each unit increase in PLR was associated with a 7% decrease in the risk of glucocorticoid resistance,^11^ whereas another found that a 1-unit increase in PLR was associated with a 13% decrease in ITP recurrence^12^, highlighting an opposite prognostic trend compared with other diseases. In our study, the decrease in PLR may not indicate a true biologic or immune mechanism but may instead simply reflect the lower platelet counts in PSAIG-positive dogs, because no significant difference in lymphocyte numbers was observed between groups. Additional studies are needed to evaluate whether PLR has diagnostic or prognostic utility in dogs with suspected ITP.

Our study had some limitations. Firstly, although thrombocytopenic dogs were classified based on comorbid conditions, the sample size within each disease category, along with the limited number of PSAIG-positive cases (*n* = 16), was too small to permit logistic regression analysis of their individual impact on PSAIG positivity. Secondly, we did not perform an a priori sample size calculation, and the possibility of a Type II error cannot be excluded. This lack of statistical power is central to interpreting the findings for PLR. The discrepancy in statistical significance for PLR between automated and manual platelet counts can be explained by the small effect size observed in our cohort, as detailed in Table 2. The univariable logistic regression analysis showed that the ORs for PLR were very close to 1.0 for both automated (OR, 0.94) and manual (OR, 0.97) counts, indicating a weak association with PSAIG positivity. For the manual PLR, the 95% CI (0.95-1.00) included 1.0, meaning a true lack of association could not be excluded, rendering the result not significant. The fact that the automated PLR was significant, whereas the manual PLR was not, despite having nearly identical ORs, highlights the fragility of a *P*-value near the .05 threshold in an underpowered study. These data, also visualized in Figure 1, suggest the effect size was small. Given that the analysis relied on univariate regression, the findings are unstable and do not account for potential confounders. Therefore, the clinical relevance of PLR as a predictor for PSAIG status in this context should be interpreted cautiously. Thirdly, not all dogs underwent a comprehensive diagnostic evaluation for all potential causes of thrombocytopenia. As a result, the possible presence of undiagnosed underlying conditions cannot be excluded and may have contributed to PSAIG positivity in some cases. This limitation should be considered when interpreting the association between PSAIG and vector-borne diseases in our study. Fourthly, as mentioned above, our study did not include PCR testing for *Anaplasma* spp. The detection of *Anaplasma* spp. antibodies may reflect prior exposure rather than active infection, adding complexity to the interpretation of our results. Future studies incorporating PCR testing would provide additional insight into the association between active infection in PSAIG development. Lastly, our study focused on a single geographical region, which may restrict the generalizability of the findings to other dog populations with different demographics or environmental exposures.

In conclusion, our study found that lower platelet counts and PLR calculated using the automated platelet count were significantly associated with PSAIG positivity, reinforcing the potential link between PSAIG and thrombocytopenia severity. Among vector-borne pathogens, *E. canis* infection, particularly when accompanied by *Anaplasma* spp. seropositivity was associated with the highest odds of PSAIG positivity, supporting a possible immune-mediated mechanism in co-infected or co-exposed dogs.

## Off-label antimicrobial declaration

Authors declare no off-label use of antimicrobials.

## Institutional animal care and use committee or other approval declaration

Approved by the Faculty of Veterinary Science Animal Care and Use Committee under protocol number MUVS-February 6, 2022.

## Abbreviations

CME: canine monocytic ehrlichiosis
ITP: immune thrombocytopenia
PSAIG: platelet surface-associated immunoglobulin
TPO: thrombopoietin
